# Pharmacokinetics, Safety, and Clinical Outcomes of Omadacycline in Women with Cystitis: Results from a Phase 1b Study

**DOI:** 10.1128/AAC.02083-18

**Published:** 2019-04-25

**Authors:** J. Scott Overcash, Pouru Bhiwandi, Lynne Garrity-Ryan, Judith Steenbergen, Stephen Bai, Surya Chitra, Amy Manley, Evan Tzanis

**Affiliations:** a eStudySite, San Diego, California, USA; b Wake Research, Raleigh, North Carolina, USA; c Paratek Pharmaceuticals, Inc., King of Prussia, Pennsylvania, USA

**Keywords:** cystitis, omadacycline, urinary tract infection, women

## Abstract

Omadacycline, an aminomethylcycline antibiotic, is approved as once-daily intravenous (i.v.) and oral (p.o.) monotherapy for acute bacterial skin and skin structure infections and for community-acquired bacterial pneumonia, and it is under development for treatment of urinary tract infection (UTI). This is a phase 1b, randomized, open-label study of omadacycline in women with cystitis (defined as UTI symptoms and a positive urine leukocyte esterase test). Patients received omadacycline for 5 days (group 1: 200 mg intravenously on day 1, then 300 mg orally every 24 h [q24h]; group 2: 300 mg orally every 12 h [q12h] on day 1, then 300 mg orally q24h; group 3: 450 mg orally q12h on day 1, then 450 mg orally q24h). Blood and urine samples were collected over 5 days. Investigator-assessed clinical response was determined at end of treatment (EOT; day 6) and posttreatment evaluation (PTE; 5 to 9 days after last dosing). A total of 31 women were treated. At steady state (day 5), the range of mean omadacycline urine concentrations over 24 h across the groups was 17.94 to 48.12 μg/ml. The most common treatment-emergent adverse events were gastrointestinal (including nausea [60% to 73%] and vomiting [20% to 40%]) and were generally mild and transient. Investigator-determined clinical success was observed in 94% and 84% of patients at EOT and PTE, respectively, with similar results across groups. A favorable microbiological response at PTE was observed in 78% of patients who had a baseline pathogen. Omadacycline is partially excreted in urine and appears to be safe and well tolerated. These preliminary results indicate that omadacycline warrants further evaluation in larger controlled UTI studies.

## Abstract (precis)

Omadacycline, an aminomethylcycline antibiotic, is approved as once-daily intravenous (i.v.) and oral (p.o.) monotherapy for acute bacterial skin and skin structure infections and for community-acquired bacterial pneumonia, and it is under development for treatment of urinary tract infection (UTI). This is a phase 1b, randomized, open-label study of omadacycline in women with cystitis (defined as UTI symptoms and a positive urine leukocyte esterase test).

## INTRODUCTION

Urinary tract infection (UTI) poses a substantial problem in community- and hospital-based settings; some 150 million UTIs occur worldwide each year, accounting for ∼$6 billion in health care expenditures, as reported by the American Urological Association (AUA) (https://www.auanet.org/education/adult-uti.cfm). UTIs are particularly common in women and the elderly. Among young, healthy women, nearly one in three will have had at least one episode of UTI requiring antimicrobial therapy by the age of 24 years, and almost one-third of these women will develop a second infection within 6 months of initial diagnosis (1, 2). The annual estimated incidence of UTI in premenopausal women in the United States is 0.5 to 0.7 infections/person/year, and among Medicare beneficiaries aged ≥65 years, UTIs account for 1.8 million office visits each year (https://www.auanet.org/education/adult-uti.cfm). UTIs are often categorized clinically as uncomplicated (e.g., cystitis in women with no known urological abnormalities) or complicated (e.g., pyelonephritis or UTI associated with an indwelling urinary catheter or structural urological abnormalities). The majority of community-acquired UTIs manifest as uncomplicated bacterial cystitis and mainly affect women (https://www.auanet.org/education/adult-uti.cfm). Uncomplicated UTI (uUTI) is most commonly caused by Escherichia coli (∼80% of cases) and Staphylococcus saprophyticus (∼5% to ∼15% of cases), and substantial increases in antimicrobial resistance rates in Escherichia coli have been reported (3). Based on an analysis of changes in antimicrobial resistance patterns in the United States between 2000 and 2010 (*n* = 12,253,679), the largest increases in E. coli resistance were reported for ciprofloxacin (3.0% to 17.1%) and trimethoprim-sulfamethoxazole (17.9% to 24.2%) (3).

Omadacycline is approved as a once-daily intravenous (i.v.) and oral (p.o.) antibiotic for use as empirical monotherapy in acute bacterial skin and skin structure infections (OASIS-1 study [ClinicalTrials.gov registration no. NCT02378480] [4] and OASIS-2 study [registration no. NCT02877927]) and community-acquired bacterial pneumonia (OPTIC study [registration no. NCT02531438] [5]). Omadacycline, the first aminomethylcycline antibiotic, is a semisynthetic tetracycline derivative that exhibits activity against Gram-positive and Gram-negative aerobes, anaerobes, and atypical bacteria (6). It has been shown to be active against tetracycline-susceptible and tetracycline-resistant strains (7). The MICs of omadacycline for at least 90% of isolates (MIC_90_) are 0.25 μg/ml for *S. saprophyticus* and 2 μg/ml for E. coli (8), regardless of the presence of extended-spectrum beta-lactamases (ESBLs) (9; M. D. Huband, unpublished data). A phase 1 absorption, distribution, metabolism, and excretion study in healthy human volunteers demonstrated partial renal excretion of omadacycline (10).

The primary objective of the current study, which is the first study of omadacycline in patients with UTI, was to evaluate the plasma and urine pharmacokinetics (PK) of omadacycline in women with cystitis. Safety was a secondary objective, and clinical efficacy was an exploratory objective. The results of this study were intended to determine the potential utility of omadacycline for the treatment of UTI and to aid in the selection of dosing regimens for use in possible future UTI studies.

## RESULTS

### Patient disposition.

Between May and September 2016, a total of 31 patients (11 in group 1 and 10 each in groups 2 and 3) were randomized and received study medication for 5 days at three study sites in the United States. Patients in group 1 received 200 mg i.v. on day 1, followed by 300 mg p.o. every 24 h [q24h]. Patients in group 2 received 300 mg p.o. every 12 h [q12h] on day 1, followed by 300 mg p.o. q24h; and patients in group 3 received 450 mg p.o. q12h on day 1, followed by 450 mg p.o. q24h. In line with the reduced oral bioavailability of omadacycline when administered with food, patients in this study were fasted of food and drink (except water) for at least 6 h prior to oral dosing and for 2 h after oral dosing; patients also had no dairy products, antacids, or multivitamins for 4 h after oral dosing (11). All but one patient completed the intended 5 days of study treatment (one patient in group 1 withdrew consent on study day 2 and did not complete the day 5 PK assessment or end of treatment visit).

### Baseline demographics and disease characteristics.

Patient demographics and disease characteristics were well balanced (Table 1). Patients ranged in age from 19 to 75 years (median, 38 years). Patients were generally in good health otherwise, without significant comorbidities that would affect study participation. A total of nine (29.0%) patients were postmenopausal, and three (9.7%) had diabetes mellitus. Baseline cystitis symptoms were reported as moderate or severe by most patients. No patients had a fever (temperature of >100.4°F) at baseline or during the study. At baseline, 27 (87.1%) patients had growth of one or more bacterial organisms in urine cultures, and 18 (58.1%) had one or more bacterial species that met the definition of a urinary pathogen (≥1 × 10^5^ CFU/ml in urine and considered a potential cause of UTI; Table 2). The most common urinary pathogens at baseline were E. coli (55.6%), Proteus mirabilis (22.2%), and Klebsiella pneumoniae (16.7%); *S. saprophyticus* was detected in one patient (5.6%). There were no patients with positive blood cultures in this study.

**TABLE 1:** Demographics and baseline characteristics (safety population)

Parameter	Data by patient group (omadacycline dose)^*a*^:			
	1 (i.v. 200 mg → p.o. 300 mg [*n* = 11])	2 (p.o. 300 mg [*n* = 10])	3 (p.o. 450 mg [*n* = 10])	All patients (*N* = 31)
Race (*n* [%])				
White	4 (36.4)	3 (30.0)	4 (40.0)	11 (35.5)
Black or African American	6 (54.5)	7 (70.0)	6 (60.0)	19 (61.3)
Asian	1 (9.1)	0	0	1 (3.2)
Female (*n* [%])	11 (100.0)	10 (100.0)	10 (100.0)	31 (100.0)
Age (yrs) (median [range])	51 (20–60)	31 (25–54)	41 (19–75)	38 (19–75)
Wt (kg) (median [range])	72 (47–95)	75 (54–121)	69 (51–111)	72 (47–121)
Body mass index (kg/m^2^) (median [range])	27 (20–39)	28 (20–43)	27 (21–41)	27 (20–43)
Medical history (*n* [%])				
Postmenopausal	5 (45.5)	1 (10.0)	3 (30.0)	9 (29.0)
Diabetes mellitus	0	1 (10.0)	2 (20.0)	3 (9.7)

a Omadacycline doses are given in parentheses after each group. i.v., intravenous; p.o., oral.

**TABLE 2:** Baseline pathogens (safety population)

Parameter or pathogen^*b*^	Data by patient group (omadacycline dose)^*a*^:			
	1 (i.v. 200 mg → p.o. 300 mg [*n* = 11])	2 (p.o. 300 mg [*n* = 10])	3 (p.o. 450 mg [*n* = 10])	All patients (*N* = 31)
Patients with culture growth at baseline (*n* [%])	8 (72.7)	9 (90.0)	10 (100.0)	27 (87.1)
Patients with pathogen growth at baseline^*c*^ (*N*1 [%])	6 (54.5)	5 (50.0)	7 (70.0)	18 (58.1)
Patients with Gram-negative pathogens^*d*^ (*n* [%])	6 (100.0)	4 (80.0)	6 (85.7)	16 (88.9)
Escherichia coli	3 (50.0)	3 (60.0)	4 (57.1)	10 (55.6)
Klebsiella pneumoniae	0	1 (20.0)	2 (28.6)	3 (16.7)
Proteus mirabilis	3 (50.0)	1 (20.0)	0	4 (22.2)
Patients with Gram-positive pathogens^*d*^ (*n* [%])	1 (16.7)	1 (20.0)	2 (28.6)	4 (22.2)
Aerococcus urinae	0	0	1 (14.3)	1 (5.6)
Enterococcus faecalis	1 (16.7)	0	0	1 (5.6)
Staphylococcus saprophyticus	0	0	1 (14.3)	1 (5.6)
Streptococcus agalactiae (group B)	0	1 (20.0)	0	1 (5.6)

a Omadacycline doses are given in parentheses after each group. i.v., intravenous; p.o., oral.

b Baseline pathogens were defined as bacteria identified in urine at ≥1 × 10^5^ CFU/ml and considered a potential cause of urinary tract infection. Patients may have had more than one pathogen. *N*1, number of subjects with organism growth at baseline.

c Percentages were calculated as 100 × (*N*1/*N*).

d Percentages were calculated as 100 × (*n*/*N*1).

### Pharmacokinetics.

**FIG 1:**
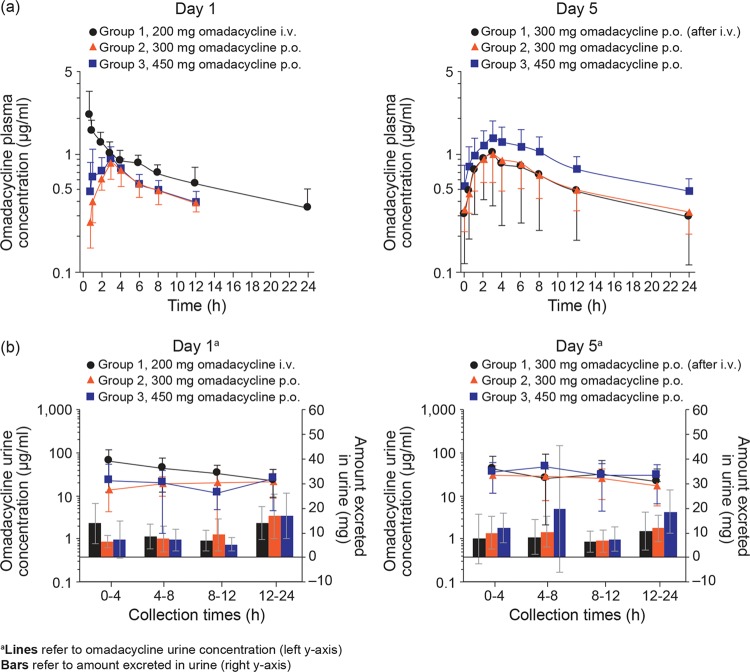
(a) Mean (±standard deviation) concentrations of omadacycline in plasma; (b) mean (±standard deviation) concentrations of omadacycline and amount excreted in urine (pharmacokinetic population).

Steady-state concentrations of omadacycline were achieved by the end of day 1 using either a single i.v. dose of 200 mg or a p.o. dose of 300 mg or 450 mg every 12 h (Fig. 1a).

After a single 200-mg i.v. dose of omadacycline, the mean maximum (peak) observed plasma concentration (*C*_max_) was 2.16 μg/ml (Table 3). The area under the concentration-time curve (AUC) from 0 to 24 h (AUC_0–24_) for i.v. omadacycline was 16.02 μg · h/ml on day 1. The mean clearance (CL) and volume of distribution (*V*) values were 10.27 liters/h and 167.57 liters, respectively. Using the omadacycline p.o. loading dose paradigm on day 1, the mean *C*_max_ and AUC from 0 to 12 h for 450 mg every 12 h (0.99 μg/ml and 6.85 μg · h/ml, respectively) were only modestly higher than for 300 mg every 12 h (0.88 μg/ml and 6.26 μg · h/ml). At steady state (day 5), *C*_max_ and AUC_0–24_ values in patients in groups 1 and 2 (both receiving 300 mg p.o. once daily) varied by only approximately ∼3% (*C*_max_ was 1.12 μg/ml in both groups; AUC_0–24_ was 13.16 μg · h/ml in group 1 and 13.50 μg · h/ml in group 2), whereas in group 3 (450 mg p.o. once daily), the *C*_max_ and AUC_0–24_ values (1.49 μg/ml and 19.83 μg · h/ml, respectively) were ∼33% and ∼46% higher, respectively, compared with values in groups 1 and 2. The median times to reach *C*_max_ (*T*_max_) were 0.75 h for the i.v. dose and 3.0 h for the p.o. doses (Table 3).

**TABLE 3:** Mean omadacycline pharmacokinetics (PK) in plasma and urine (PK population)

Parameter	Results by group on day^*a*^:					
	1			5		
	Group 1 (i.v. 200 mg → p.o. 300 mg [*n* = 11])	Group 2 (p.o. 300 mg [*n* = 10])	Group 3 (p.o. 450 mg [*n* = 10])	Group 1^*b*^ (i.v. 200 mg → p.o. 300 mg [*n* = 10])	Group 2 (p.o. 300 mg [*n* = 10])	Group 3 (p.o. 450 mg [*n* = 10])
Plasma^*c*^						
*C*_max_ (μg/ml)	2.16 (59.0)	0.88 (25.6)	0.99 (30.6)	1.12 (58.8)	1.12 (37.2)	1.49 (36.2)
*T*_max_ (h)	0.75 (0.72, 1.60)	3.0 (2.0, 4.0)	3.0 (0.97, 4.0)	3.0 (0.0, 4.0)	3.0 (2.0, 8.0)	3.0 (0.50, 8.0)
AUC_0–24_ (μg · h/ml)	16.02 (28.7)	ND	ND	13.16 (59.7)	13.50 (33.7)	19.83 (30.8)
AUC_0–12_ (μg · h/ml)	ND	6.26 (18.9)	6.85 (23.2)	ND	ND	ND
*T*_1/2_ (h)	11.38 (13.6)	ND	ND	ND	ND	ND
Clearance (liters/h)	10.27 (15.1)	ND	ND	ND	ND	ND
*V* (liters)	167.57 (17.6)	ND	ND	ND	ND	ND
*C*_min_ (μg/ml)	ND	ND	ND	0.29 (60.4)	0.32 (35.4)	0.48 (27.6)
Urine^*d*^						
Ae_0–24_ (mg)^*e*^	40.70 (44)	39.24 (34)	35.95 (33)	32.27 (79)	38.21 (52)	54.87 (67)
Total dose within 24 h (mg) (delivery method)	200 (i.v.)	600 (p.o.)	900 (p.o.)	300 (p.o.)	300 (p.o.)	450 (p.o.)
fe_0–24_ (%) (dose administered)	20.4	6.5	4.0	10.8	12.7	12.2
Concentration (μg/ml) at time^*f*^						
0–4 h	65.36 (53.02)	14.00 (9.69)	23.01 (29.94)	42.71 (43.37)	30.90 (18.90)	35.99 (24.12)
4–8 h	43.55 (31.52)	19.09 (9.47)	20.54 (19.19)	26.09 (18.06)	29.11 (21.37)	48.12 (46.01)
8–12 h	32.41 (20.32)	19.98 (19.23)	11.70 (6.82)	33.24 (34.55)	25.35 (16.99)	30.47 (25.84)
12–24 h	22.41 (17.75)	20.77 (11.92)	25.71 (16.24)	21.48 (21.97)	17.94 (12.00)	30.28 (23.57)
CL_R_^*g*^ (liters/h)^*f*^	2.64 (1.21)	ND	ND	2.42 (0.92)	2.71 (1.01)	2.80 (2.29)

a Omadacycline doses are given in parentheses after each group. i.v., intravenous; p.o., oral; ND, not determined.

b One patient in group 1 withdrew consent on study day 2 and did not complete the day 5 PK assessment.

c Arithmetic mean (percent coefficient of variation [%CV]) is shown for all parameters except *T*_max_, which is shown as median and range (minimum, maximum). *C*_max_, mean maximum (peak) observed plasma concentration; *T*_max_, median time to maximum (peak) observed plasma concentration; AUC_0–24_, area under concentration-time curve from 0 to 24 h.; AUC_0–12_, area under the concentration-time curve from 0 to 12 h; *T*_1/2_, half-life; *V*, volume of distribution; *C*_min_, mean minimum observed plasma concentration.

d Ae_0–24_, amount excreted unchanged in urine from 0 to 24 h; fe_0–24_, fraction of dose excreted in urine from 0 to 24 h.

e Data shown are mean (%CV).

f Data shown are mean (standard deviation [SD]).

g CL_R_, renal clearance.

The highest omadacycline concentration in urine (mean, 65.4 μg/ml) was observed in group 1 between 0 and 4 h after administration of the 200-mg i.v. dose on day 1 (Fig. 1b). Mean values across all other groups and time intervals ranged from 11.7 μg/ml to 48.1 μg/ml. After dosing on day 1, mean values for the cumulative amounts of drug excreted unchanged in urine from 0 to 24 h (Ae_0–24_) were similar in each group (35.95 to 40.70 mg; Table 3). At steady state (day 5), the mean urine concentration for 0 to 24 h ranged from 17.94 to 48.12 μg/ml across the 3 groups; mean Ae_0–24_ values were similar in groups 1 and 2 (32.27 mg and 38.21 mg), following administration of 300 mg p.o. once daily, and higher in group 3 (54.87 mg), following administration of 450 mg p.o. once daily. Mean values for the fraction of the dose excreted unchanged in urine from 0 to 24 h after dosing (fe_0–24_) at steady state (day 5) ranged from 10.8% to 12.7%. After the p.o. dose on day 5, mean renal clearance values were also similar in each group (range, 2.42 liters/h to 2.80 liters/h).

### Efficacy.

Patient-reported clinical success at end of treatment (EOT) occurred in 72.7% of patients in group 1, 100% in group 2, and 90.0% in group 3. Investigator-determined clinical success was ≥90.0% in all groups at EOT (Table 4). At posttreatment evaluation (PTE), these rates were 100% in group 1, 70.0% in group 2, and 80.0% in group 3. Four patients had clinical failure across groups 2 and 3, among whom two patients had no known baseline pathogens, one patient had K. pneumoniae (omadacycline MIC = 2 μg/ml) with a favorable microbiological response and *A. urinae* (omadacycline MIC ≤ 0.06 μg/ml) with a favorable microbiological response, and one patient had E. coli (omadacycline MIC = 1 μg/ml) with an unfavorable microbiological response and P. mirabilis (omadacycline MIC = 8 μg/ml) with a favorable microbiological response. Among patients with a baseline pathogen, favorable microbiological responses at PTE occurred in 100% of patients in group 1, 80.0% in group 2, and 57.1% in group 3 (Table 5). Four patients had unfavorable microbiological responses across groups 2 and 3, three patients had baseline E. coli with MIC values of 0.5 to 1 μg/ml, and one patient had K. pneumoniae with an MIC of 2 μg/ml. Data for favorable microbiological response and clinical success at PTE by baseline pathogen and MIC are provided in Table 6.

**TABLE 4:** Investigator assessment of clinical response to omadacycline (safety population)

Parameter^*b*^	No. (%) of patients in group^*a*^:		
	1 (i.v. 200 mg → p.o. 300 mg [*n* = 11])	2 (p.o. 300 mg [*n* = 10])	3 (p.o. 450 mg [*n* = 10])
End of treatment			
Clinical success	10 (90.9)	10 (100)	9 (90.0)
Clinical failure	0	0	1 (10.0)
Indeterminate	1 (9.1)	0	0
Posttreatment evaluation			
Clinical success	11 (100)	7 (70.0)	8 (80.0)
Clinical failure	0	2 (20.0)	2 (20.0)
Indeterminate	0	1 (10.0)	0

a Omadacycline doses are given in parentheses after each group. i.v., intravenous; p.o., oral.

b Clinical success indicates resolution of signs and symptoms and no use of additional systemic antimicrobial therapy for the urinary tract infection. Clinical failure indicates no apparent response to therapy, persistence of signs and symptoms of infection at the end-of-treatment visit, or the use of additional systemic antimicrobial therapy for the current infection. Indeterminate indicates that the visit was not completed.

**TABLE 5:** Microbiological response at the posttreatment evaluation visit in patients with a baseline pathogen (safety population)

Parameter	Data by patient group (omadacycline dose)^*a*^:		
	1 (i.v. 200 mg → p.o. 300 mg [*n* = 11])	2 (p.o. 300 mg [*n* = 10])	3 (p.o. 450 mg [*n* = 10])
Patients with pathogen growth at baseline (*N*1 [%])^*b*^	6 (54.5)	5 (50.0)	7 (70.0)
Outcome (*n* [%])^*c*^			
Favorable^*d*^	6 (100.0)	4 (80.0)	4 (57.1)
Unfavorable	0	1 (20.0)	3 (42.9)
Indeterminate	0	0	0

a Omadacycline doses are given in parentheses after each group. i.v., intravenous; p.o., oral.

b Percentages were calculated as 100 × (*N*1/*N*). *N*1, number of subjects with organism growth at baseline; *N*, total number of subjects.

c Favorable indicates that each baseline pathogen was reduced to <10^4^ CFU/ml. Unfavorable indicates that at least one baseline pathogen was not reduced to <10^4^ CFU/ml. Indeterminate indicates that follow-up cultures were not obtained.

d Percentages were calculated as 100 × (*n*/*N*1).

**TABLE 6:** Favorable microbiological response and investigator-assessed clinical success at the posttreatment evaluation visit

Baseline pathogen	Omadacycline MIC (μg/ml)	Favorable microbiological response at PTE^*a*^ (*n*/*N* [%])	Clinical success^*b*^ at PTE (*n*/*N* [%])
Gram-negative pathogens			
Escherichia coli	Overall^*c*^	7/10 (70.0)	9/10 (90.0)
	0.5	2/3 (66.7)^*d*^	3/3 (100.0)
	1.0	4/6 (66.7)^*e*^^,^^*f*^	1/6 (16.7)^*d*^
	2.0	1/1 (100.0)	1/1 (100)
Klebsiella pneumoniae	Overall^*c*^	2/3 (66.7)	2/3 (66.7)
	1.0	1/1 (100.0)	1/1 (100.0)
	2.0	1/2 (50.0)^*f*^	1/2 (50.0)^*g*^
Proteus mirabilis	Overall^*c*^	4/4 (100.0)	3/4 (75.0)
	8.0	2/2 (100.0)	1/2 (50.0)^*e*^
	16.0	2/2 (100.0)	2/2 (100.0)
Gram-positive pathogens			
Aerococcus urinae	≤0.06	1/1 (100.0)	0/1 (0)^*g*^
Enterococcus faecalis	≤0.06	1/1 (100.0)	1/1 (100.0)
Staphylococcus saprophyticus	0.25	1/1 (100.0)	1/1 (100.0)
Streptococcus agalactiae (group B)	0.12	1/1 (100.0)	1/1 (100.0)

a Favorable microbiological response indicates the baseline pathogen was reduced to <10^4^ CFU/ml at posttreatment evaluation (PTE). Percentages are based on the number of patients with the baseline pathogen at the specified MIC. *n*, number of patients in the specific category; *N*, number of patients with the baseline pathogen at the specified MIC.

b Clinical success indicates resolution of signs and symptoms and no use of additional systemic antimicrobial therapy for the urinary tract infection; clinical failure indicates no apparent response to therapy, persistence of signs and symptoms of infection at the end-of-treatment visit, or the use of additional systemic antimicrobial therapy for the current infection. Percentages are based on the number of patients with the baseline pathogen at the specified MIC.

c “Overall” indicates all isolates of the same pathogen species across the MIC distribution.

d One patient in group 3 had an unfavorable microbiological response at PTE.

e One patient in group 2 had E. coli with an unfavorable microbiological response at PTE and P. mirabilis with a favorable microbiological response at PTE. The patient was assessed as a clinical failure at PTE.

f One patient in group 3 had an unfavorable microbiological response at PTE.

g One patient in group 3 had K. pneumoniae with a favorable microbiological response at PTE and A. urinae with a favorable microbiological response at PTE. The patient was assessed as a clinical failure at PTE.

Urinalysis was notable only for leukocyte esterase testing, which shifted from positive at baseline (trace to 3+) to a less severe category or negative at EOT for most patients. This finding is consistent with what was expected, as all patients had to have a positive leukocyte esterase test at screening to qualify for the study, and it would be expected that this would improve or become negative if the infection was improving/resolving.

### Safety.

The percentage of patients with any treatment-emergent adverse event (TEAE) was comparable in each group (80.0% to 90.0%; Table 7). The percentage of patients with treatment-related TEAEs was similar in each treatment group (group 1, 81.8%; group 2, 90.0%; group 3, 80.0%). There were no severe or serious TEAEs nor any TEAEs resulting in premature treatment discontinuation. Gastrointestinal events were the most common TEAEs in each group, most notably nausea (60.0% to 72.7%), vomiting (20.0% to 40.0%), and diarrhea (0.0% to 20.0%); all were mild or moderate in intensity and generally transient. No patients discontinued omadacycline because of these TEAEs. However, 7 (23.0%) patients received the antiemetic/antinauseant medication ondansetron during the study.

**TABLE 7:** Treatment-emergent adverse events in >1 patient (safety population)

TEAE	No. (%) of patients in group^*a*^:			
	1 (i.v. 200 mg p.o. 300 mg [*n* = 11])	2 (p.o. 300 mg [*n* = 10])	3 (p.o. 450 mg [*n* = 10])	All patients (*n* = 31)
Any TEAE^*b*^	9 (81.8)	9 (90.0)	8 (80.0)	26 (83.9)
Nausea	8 (72.7)	6 (60.0)	6 (60.0)	20 (64.5)
Vomiting	4 (36.4)	4 (40.0)	2 (20.0)	10 (32.3)
Headache	3 (27.3)	2 (20.0)	1 (10.0)	6 (19.4)
Amylase increased	0	3 (30.0)	1 (10.0)	4 (12.9)
Vulvovaginal mycotic infection	1 (9.1)	1 (10.0)	2 (20.0)	4 (12.9)
Diarrhea	0	2 (20.0)	1 (10.0)	3 (9.7)
Gastroesophageal reflux disease	1 (9.1)	0	1 (10.0)	2 (6.5)
Pruritus	2 (18.2)	0	0	2 (6.5)

a Omadacycline doses are given in parentheses after each group. i.v., intravenous; p.o., oral.

b TEAE, treatment-emergent adverse event.

Clinically asymptomatic elevations in pulse rate were observed in each treatment group. The largest median increase in pulse rate (20 beats per min) was observed in group 1 after the 200-mg i.v. dose. The largest median increase in pulse rate in groups 2 and 3 after p.o. doses of 300 mg or 450 mg was ∼15 beats per min. These changes were transient, generally occurring between 1 and 3 h postdose, and then resolving over several hours. There were no clinically relevant changes in blood pressure, 12-lead electrocardiogram parameters, or physical examination results.

Laboratory testing was notable only for a small increase from baseline in serum alanine aminotransferase (ALT) values at the end of treatment in group 3 following 450-mg p.o. dosing (median change, 18 U/liter). The corresponding median changes in ALT in groups 1 and 2 were ≤6.0 U/liter. The highest individual ALT value (92 U/liter; 2.8-fold greater than the upper limit of normal [ULN]) occurred at EOT in a patient in the 450-mg p.o. dose group, but the ALT value subsequently declined to 40 U/liter at PTE. There were no clinically meaningful changes from baseline or differences between groups in other laboratory parameters.

## DISCUSSION

This study, the first evaluation of omadacycline in patients with UTI, demonstrated that the PK of omadacycline in women with cystitis is similar to the PK previously observed in healthy subjects (10–13). In previous studies, *C*_max_ varied across doses and infusion time, with a mean of 1.8 μg/ml for 100 mg i.v. administration and 0.7 μg/ml for 300 mg oral. The mean terminal elimination half-life was 17 h, although this varied across doses, and mean clearance was 17.1 liters/h, independent of the dose used (12). In a mass balance study, 14.4% of a 300-mg oral dose of omadacycline, corresponding to ∼40% of the absorbed dose, was excreted by the kidneys and was present as active drug in the urine (10). In the current study, at steady state the fraction of omadacycline excreted in urine over 24 h after p.o. dosing was ∼12%, which, based on an established p.o. bioavailability of 35% (14), translates to ∼34% of the absorbed dose.

In the current study, both i.v. and p.o. administration of omadacycline resulted in urine concentrations that exceeded peak plasma concentrations and were sustained over the 24-h dosing interval. At steady state, the p.o. dosing regimens of 300 mg or 450 mg once daily provided mean omadacycline concentrations in urine (range, 17.94 μg/ml to 48.12 μg/ml) that compare favorably to the omadacycline MIC_90_ values for common UTI pathogens, such as E. coli (MIC_90_ = 2 μg/ml) (8) and *S. saprophyticus* (MIC_90_ = 0.25 μg/ml) (M. D. Huband, unpublished data). These data indicate that once-daily p.o. dosing can achieve concentrations of omadacycline (sustained over the 24-h dosing period) that will exceed the MIC_90_ for common UTI pathogens. The proportion of the administered p.o. dose excreted in the urine, together with the extensive tissue distribution demonstrated in earlier PK studies, suggests that omadacycline may also have clinical utility in complicated UTI, including acute pyelonephritis.

It was encouraging that exploratory analyses of efficacy demonstrated high clinical response rates, with investigator-determined clinical success in 94% and 84% of patients at EOT and PTE, respectively, across all 31 treated patients. Clinical failure and unfavorable microbiological response were not correlated, and no factor was identified that could explain the unfavorable clinical or microbiological response in all patients. However, given the small sample size and the relatively high clinical response rates, no conclusions can be drawn with respect to potential efficacy differences between the dosing regimens evaluated.

The safety profile of omadacycline in the female patients with cystitis in the current study was comparable with that observed in other study populations receiving similar omadacycline dosing regimens (6, 10, 11, 13). The only notable exception was the incidence of gastrointestinal TEAEs, especially nausea and vomiting, which was higher than expected in the current study. However, no patient required study treatment discontinuation for TEAEs. Lower incidences of nausea (8% and 4%) and vomiting (8% and 0%) had been observed in a phase 1, p.o., multidose study in healthy volunteers at 300-mg and 450-mg omadacycline doses, respectively (11). The reason for the higher incidences of nausea and vomiting in the current study is not clear, and the study design and small sample size make this challenging to interpret. The observations in some patients of transient, asymptomatic changes in heart rate or serum ALT have been observed in prior studies of omadacycline in healthy subjects (10, 11, 13).

Antibiotic rates of resistance to current standard therapies have increased for outpatient UTI E. coli isolates, and multidrug-resistant E. coli and ESBL-positive E. coli strains are being isolated from community-acquired infections more frequently than has been observed previously (3, 15). There is a particular need for new antimicrobials with appropriate coverage of UTI pathogens that are orally bioavailable and thus can be used primarily for outpatient treatment or transition from i.v. to p.o. therapy upon hospital discharge. The antibacterial spectrum of omadacycline, in particular its activity against ESBL-positive E. coli, development of both i.v. and p.o. formulations, its plasma and urine PK, and its safety and tolerability profile suggest that omadacycline has the potential to be a useful antibiotic for the treatment of UTI. As this was a small-scale study, the findings are limited by the small number of patients included and by associated variation in PK parameters. The observed variation in parameters such as *C*_max,_ should be used to estimate the required sample sizes for future larger-scale studies. In addition, larger studies that focus on specific UTI patient populations, such as uUTI and complicated UTI (i.e., acute pyelonephritis), and/or a specific spectrum of bacterial pathogens, are needed to better understand the potential role and appropriate dose of omadacycline for the treatment of UTI.

## MATERIALS AND METHODS

### Patient population.

Adult (≥18 years of age) women who had an onset of ≥2 cystitis symptoms (dysuria, urgency, frequency, or suprapubic pain) within ≤72 h before randomization were eligible. Patients were required to have a positive urine leukocyte esterase test and to provide a clean-voided midstream urine sample for microbiological analysis at screening. Patients were excluded for a known or suspected complicated UTI, previous treatment with a systemic antibiotic ≤48 h before randomization, history or evidence of severe renal disease or a calculated creatinine clearance of <30 ml/min using the Cockcroft-Gault equation, or the need for any form of dialysis (e.g., hemodialysis or peritoneal dialysis). Also excluded were patients with a known history of unstable cardiac disease (e.g., unstable angina, myocardial infarction, acute congestive heart failure, or unstable cardiac arrhythmia) within 3 months before screening. Additionally, patients with elevated levels of ALT (≥3 × ULN), aspartate aminotransferase (≥3 × ULN), or total bilirubin (>1.5 × ULN) were not eligible. Complete study entry criteria are provided in the Supplemental Material.

The study was designed, conducted, and reported in accordance with the ethical principles laid down in the Declaration of Helsinki. All patients provided written informed consent prior to participation. The study was approved by a properly constituted institutional review board/independent ethics committee/research ethics board (Quorum Review IRB, Seattle, WA, USA) before study start. The patients were enrolled from 1 May 2016 (first patient enrolled) to 26 September 2016 (last patient completed).

### Study design.

In this phase 1b, open-label, randomized (1:1:1), parallel-group study, patients received one of three omadacycline regimens for 5 days (Fig. S1 in the Supplemental Material). All patients were to remain housed at the clinical site from the start of treatment (day 1) through the EOT assessment on day 6.

### Dose selection.

Previous clinical data suggested that 300 mg was the lowest daily p.o. dose of omadacycline that would maintain favorable urine concentrations to treat common uUTI pathogens (10). The omadacycline half-life of ∼18 h suggested that once-daily dosing would be justified (10). Given this half-life, between 72 and 90 h would be required to achieve steady-state conditions at a constant dosing rate. To reduce the time required to achieve target concentrations, a “loading dose” strategy was employed. Group 1 used an initial dose of 200 mg i.v. on day 1, followed by once-daily doses of 300 mg p.o. The 300-mg p.o. dose is bioequivalent to a 100-mg i.v. dose; hence, 200 mg i.v. was expected to be comparable with 600 mg p.o. Because early studies demonstrated an increased incidence of gastrointestinal adverse events (AEs) at doses of ≥400 mg p.o. (12), the 200-mg i.v. dose was evaluated as a potential means of providing a rapid and better tolerated “loading dose.” Group 2 used two doses of 300 mg p.o. (separated by 12 h) on day 1, followed by once-daily doses of 300 mg p.o. Group 3 evaluated higher doses of omadacycline—initial dosing with two doses of 450 mg p.o. (separated by 12 h) on day 1, followed by once-daily doses of 450 mg p.o.—with the goal of achieving a greater exposure that may be required in future studies. Based on prior phase 1 studies, the 450-mg p.o. dose was considered near the upper end of the dose range for providing acceptable tolerability in treating UTI (11).

### Study assessments.

Blood and urine samples were collected on days 1 and 5 for PK and safety analyses. Plasma and urine samples were analyzed for determination of omadacycline concentrations using a validated liquid chromatography-mass spectrometry/mass spectrometry analytical method at Q2 Solutions (formerly Quintiles BioSciences; Ithaca, New York, USA). Safety assessments included adverse events, 12-lead electrocardiogram recordings, clinical laboratory testing, vital sign measurements, and physical examinations. Efficacy assessments consisted of investigator-assessed clinical response and microbiologic outcome at EOT (day 6) and at PTE (5 to 9 days after the last dose). Urine cultures were performed at baseline, daily through EOT, and at PTE. Cystitis symptoms (including frequency, urgency, pain, incomplete voiding, fever, hypothermia, and blood in urine) were assessed by patients using the Urinary Tract Infection Symptoms Assessment questionnaire (16).

### Statistical analysis.

The safety population comprised all randomized patients who received any amount of omadacycline; the PK population comprised all randomized patients who received omadacycline and had evaluable PK parameter data.

Descriptive statistics were presented for the PK parameter estimates for AUC, *C*_max_, and *T*_max_ after treatment on days 1 and 5. The concentration, amount excreted in urine over a dosing interval, urinary excretion rate, and renal clearance of omadacycline after day 1 and day 5 doses were summarized by treatment group.

Adverse events were coded using Medical Dictionary for Regulatory Activities terminology. The incidence of TEAEs was presented by system organ class and preferred term, relationship to study medication, severity, and those that were serious or led to premature treatment discontinuation. Descriptive statistics for continuous-variable safety parameters were provided for each treatment group by visit and change from baseline.

The number and percentage of patients with investigator-assessed outcomes of “clinical success,” “clinical failure,” and “indeterminate” were presented for EOT and PTE by treatment group. Clinical success was defined as resolution of signs and symptoms of UTI at the EOT visit and no use of additional systemic antimicrobial therapy for the UTI. Clinical failure was defined as the persistence (or reappearance) of signs and symptoms, or use of additional systemic antimicrobial therapy for the UTI. If the EOT or PTE visit was not completed, the response was considered indeterminate. Patient-reported clinical response at EOT was programmatically determined; clinical success was defined as improvement from baseline by at least one number score in at least two of the key symptoms (dysuria, frequency, urgency, and suprapubic pain), with no worsening in any of the other symptoms.

Microbiological outcomes at PTE were defined as eradication, persistence, or indeterminate. Eradication indicated that each baseline pathogen found at ≥10^5^ CFU/ml in urine was reduced to <10^4^ CFU/ml and was not present in a blood culture (17). Microbiological failures did not meet that criterion and, if no follow-up urine culture was obtained, the response was considered indeterminate.

## Supplementary Material

Supplemental file 1
