# Supplementary material for: Pharmacokinetics, Safety, and Clinical Outcomes of Omadacycline in Women with Cystitis: Results from a Phase 1b Study
Source: Antimicrob Agents Chemother. 2019 Apr 25;63(5):e02083-18. doi: 10.1128/AAC.02083-18 (PMC6496050; doi:10.1128/AAC.02083-18)
Supplement: Supplemental file 1 [file AAC.02083-18-s0001.pdf]

Supplementary Appendix

Methods

Inclusion Criteria

1. Written and signed informed consent must have been obtained before any assessment was performed
2. Females age 18 years or older
3. Had onset of TWO or more of the following clinical signs and symptoms of a urinary tract infection (UTI) within less than or equal to 72 hours prior to randomization:
   - Dysuria
   - Frequency
   - Urgency
   - Suprapubic pain
4. Collection of a clean-voided midstream urine sample for microbiological analysis at Screening
5. Positive urine dipstick test for leukocyte esterase at Screening
6. All patients must have had a negative serum pregnancy test at Screening and agreed to comply with using an acceptable form of birth control (eg, abstinence, oral [PO] contraceptive, intrauterine device [IUD], barrier contraception [condom], tubal ligation, hysterectomy, bilateral oophorectomy, postmenopausal or vasectomized partner) from Screening through PTE
7. Were able to communicate well with the investigator and to understand and comply with the requirements of the study

Exclusion Criteria

1. Males.
2. Pregnant or nursing (breastfeeding) women
3. Had received 1 or more doses of a systemic antibacterial treatment within the 48-hour period prior to randomization
4. Known or suspected renal calculi, stricture, primary renal disease (eg, polycystic renal disease), neurogenic bladder, or other anatomic or functional abnormalities predisposing to UTI
5. Had a suspected upper UTI (eg, fever with flank pain or chills)
6. Had known or was clinically suspected to have 1 or more of the following prior to randomization:
   - Alanine aminotransferase (ALT) or aspartate aminotransferase (AST) greater than or equal to 3 × upper limit of normal (ULN)
   - Total bilirubin greater than 1.5 × ULN
   - Evidence of end-stage liver disease (eg, ascites, hepatic encephalopathy)
7. Had a known history of having experienced unstable cardiac disease (eg, unstable angina, myocardial infarction, acute congestive heart failure, unstable cardiac arrhythmia) within the 3 months prior to Screening
8. Required any form of dialysis (eg, hemodialysis, peritoneal dialysis)
9. History or evidence of severe renal disease or had a calculated creatinine clearance (CrCl) of less than 30 mL/minute, using the Cockcroft-Gault equation
10. Significant immunological disease determined by any of the following:

- Current or anticipated neutropenia defined as less than 500 neutrophils/mm^3
- Known infection with human immunodeficiency virus (HIV) or other acquired immune deficiency syndrome (AIDS)-defining illness as determined by the investigator

11. The receipt of cancer chemotherapy, radiotherapy, or potent, non-corticosteroid immunosuppressant drugs (eg, cyclosporine, azathioprine, tacrolimus, immune-modulating monoclonal antibody therapy) within the past 3 months, or the receipt of corticosteroids equivalent to or greater than 40 mg of prednisone per day or for more than 14 days in the prior 30 days
12. Had a history of hypersensitivity or allergic reaction (eg, anaphylaxis, urticaria, other significant reaction) to any tetracycline (eg, minocycline, doxycycline, or tigecycline)
13. Had a history of pseudotumor cerebri or prior (within 2 weeks prior to Screening) or planned concomitant use of isotretinoin
14. Had a history of systemic lupus erythematosus or lupus-like syndrome
15. Had current evidence of pancreatitis
16. Use of other investigational drugs within 5 half-lives or 30 days prior to Screening, whichever was longer.
17. Had been previously treated with omadacycline or previously enrolled in this study.
18. Any planned medical intervention that might have interfered with the ability to comply with the study requirements or impact the interpretation of the data
19. Had any concomitant condition that, in the opinion of the investigator, was likely to interfere with evaluation of the response of the infection under study, determination of AEs, or completion of the expected course of treatment

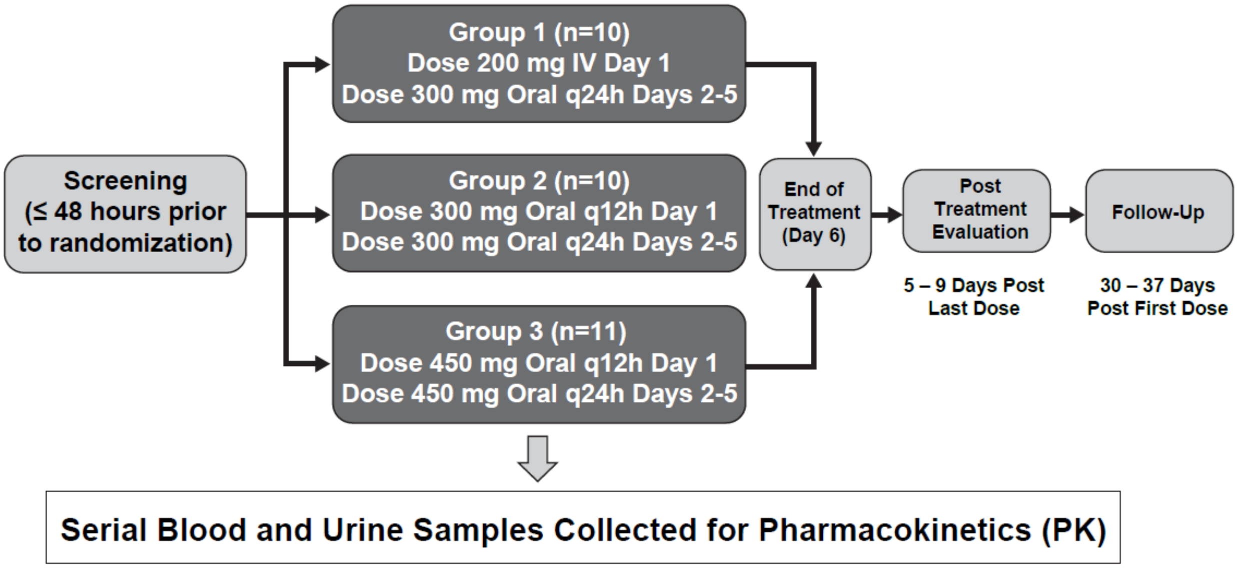

FIG S1 Study design. Abbreviations: IV, intravenous; h, hours; q, every; QD, once daily.
